# A Comparative Time-Diary Analysis of UK and US Children’s Screen Time and Device Use

**DOI:** 10.1007/s12187-021-09884-3

**Published:** 2021-12-02

**Authors:** Killian Mullan, Sandra L. Hofferth

**Affiliations:** 1 Department of Sociology and Policy, School of Social Sciences and Humanities, Aston University, Aston Triangle, Birmingham, B4 7ET UK; 2 Maryland Population Research Center, University of Maryland, College Park, MD 20742 USA

**Keywords:** Children’s time use, screen time, device use, comparative time use, UK, US

## Abstract

This paper presents the results of a comparative analysis of the time school-age children 8-17 years in the UK and the US spent using devices such as smartphones and tablets, and their time in screen-based activities such as watching TV and playing videogames in 2014-15. The paper draws on innovative instruments measuring children’s time using technology and engaging with screens in these two countries. We find that in both, children’s time using devices overlaps with time in screen-based activities, non-screen leisure, and non-leisure activities. Children in the UK spend more time using devices than children in the US, but family size and the availability of an internet connection at home largely explain major cross-national differences. Children in the US spend less time using computers than children in the UK, and, on non-school days, more time watching TV and playing videogames. These differences remain significant after controlling for a range of child, parent and family-level characteristics. Divergent cross-national patterns for children’s time using relatively new devices and their time in more established screen-based activities are linked to differences in family composition and to differential access.

## Introduction

Technological change over the past several decades has affected the daily lives of children in many countries across the world. The development and global expansion of powerful internet connectivity (OECD, 2017), alongside ready access to an increasing array of digital devices has transformed the ways in which children spend time learning, playing, consuming media, and interacting with others. A large body of research has investigated the ways in which technology has become entwined in children’s everyday lives (Bond, 2014; Kennedy & Wellman, 2007; Livingston et al., 2014; Thompson et al., 2018; Thulin & Vilhelmson, 2007; Twenge, 2017). Changing technology has influenced children’s time in screen-based activities. They now spend less time watching TV, more time playing videogames and using computers, as well as more time using smartphones and other devices during non-screen activities such as eating and travelling (Mullan, 2018; Mullan, 2020). These changes in children’s time spent in screen-based activities and in time using the internet are observed across a wide range of different countries (Bucksch et al., 2016; OECD, 2017).

There are limits, however, to what is known about the potential influence of technological change on children’s daily time use in different countries. Most prior cross-national research used recall-based measures of time use known to be unreliable. Without a common 24-hour reference period, it is impossible to combine such measures to unpack the multiple intersecting components of screen time, or reveal how time using technology intersects with time in other (non-screen) activities. As part of a comprehensive analysis of many different aspects of children’s time using 24-hour diary measures, Gracia et al. (2019) led the way in examining cross-national patterns of children’s time use (in the UK, Spain, and Finland), but they analysed only a single measure of total screen-based activities. This obscures a new distinction that has emerged between the device being used (such as the smartphone, TV, tablet, computer) and the type or function of activity engaged in (i.e., television program, movie, homework, passing time, game, e-mail, text). As Rideout (2016) pointed out, time use surveys have the potential to study time when children are engaging in screen-based activities and using devices (“media multi-tasking”), as well as time when they are using devices but not engaging in screen-based activities, such as during travel or doing homework.

Time use surveys provide reliable estimates of time in daily activities (Hofferth et al., 2008), but have been slow to adapt to recent technological change, limiting their capacity to capture fully time using new technologies (Hampton, 2007). To our knowledge, only two national time use surveys have developed instruments specifically to capture the time children use devices. These are the 2014-15 UK Time Use Survey (UKTUS) and the US Child Development Supplement 2014 (CDS) of the Panel Study of Income Dynamics (PSID). Alongside data on daily activities, both surveys allowed respondents to report any time they were using a device (e.g., smartphone, tablet, computer) regardless of what activity they were doing (see below for more details on the instruments used in these surveys). Both surveys pre-date the coronavirus pandemic and resultant lockdowns, which dramatically changed children’s daily lives, with a major shift toward spending time at home and spending it online, perhaps with long-term consequences for how children spend their time. The novel data provided by these two surveys provides an opportunity to document the “before-COVID” baseline and conduct for the first time an in-depth comparative analysis of children’s time in screen-based activities and time using devices in the UK and the US. In this paper we ask:

1. Are there differences in the time children in the UK and the US spend using devices and in screen-based activities?
2. To what extent do observed factors associated with time in these domains explain cross-national patterns, and are associations similar in the UK and the US?

## A Comparison of Children’s Technology Use in the UK and the US: What Factors Influence Use Across Countries?

In many respects, the UK and the US are similar. Although cross-national time use research typically studies countries with major linguistic, political and social differences, nevertheless, there can be much to learn from an in-depth comparison of similar countries (Livingstone, 2003), particularly when considering the influence of relatively new technologies. It is worthwhile to examine similarities–not only differences–across countries; even minor differences in key factors could have a bearing on children's time using technology in the UK and the US. Here we review cross-national patterns in children’s access to technology and the internet, parental mediation and restrictions on children's use of technology. We profile cross-national differences in factors known to influence children’s screen time and technology use, focusing on connections between these factors and children’s access to and parental control over technology, and consider the potential influence of wider societal norms and values.

Starting with internet access, available household survey data confirm that access is high in both countries, but lower in the US than in the UK. In the UK, 97% of households with children aged up to 15 years had the internet at home in 2015 (ONS, 2015). Madden et al. (2013) report that most teenagers (12-17 years) in the US can access the internet (95%), but they do not specify the proportion who have access to an internet connection at home. Lenhart (2015) reports that 90% of teenagers (13-17 years) in the US reported accessing the internet using a smartphone. Children and teenagers in the US may therefore be relatively more reliant on internet access via limited smartphone data plans or publicly accessible Wi-Fi, whereas almost all children in the UK have access to a fixed internet connection at home. Reflecting this, data from the two surveys used in this paper shows that access to the internet at home for children 8-17 years is higher in the UK than in the US (98% and 90% respectively).

Regarding devices, around 90% of children in the UK (8-15 years) and the US (8-18 years) have access to a computer or laptop at home, and around 80% have access to a tablet (Ofcom, 2015; Rideout, 2015). Tablet ownership is higher for children 8-12 years in the US (53%) compared with children 8-11 years in the UK (43%), whereas 37% of US teens (13-18 years) own a tablet compared with 45% of children 12-15 years in the UK (Ofcom, 2015; Rideout, 2015). Rates of smartphone ownership are comparatively similar, however, with around 25% of children 8-11 years (8-12 years in the US), and close to 70% of older teenage children owning a smartphone in both countries (Ofcom, 2015; Rideout, 2015). Thus, the available data suggest that children’s access to computers and digital devices is broadly similar in the UK and the US, though there are diverging patterns with respect to ownership of, but not access to, tablets in these countries.

These general access statistics mask crucial differences between groups within each country. In both the US (Lenhart, 2015; Rideout, 2015) and the UK (Ofcom, 2015), children in lower socio-economic groups have less access to devices such as laptops and tablets, and to a fixed internet connection at home, an enduring digital divide exposed by the COVID-19 pandemic. The UK and the US are both advanced wealthy economies (both are members of the G7 largest global economies), but more children live in poverty in the US than in the UK. In 2015, around 20% of US children lived in poverty compared with 11% of UK children (OECD Income Distribution Database^1^). In the US, low-income families are less likely to have a fixed internet connection at home, likely to underpin the difference in access to the internet at home noted above. To the extent that the digital divide is more pronounced, or widespread, in the US than in the UK, we might expect that US children spend less time using computers or other devices than their UK counterparts, concentrated particularly among lower socio-economic groups.

In addition to access, parental mediation and regulation of technology use can impact children’s screen time and device time. Parents may influence children’s screen time by talking with them about what they are doing online in general or about online content. Anderson (2016) finds that a vast majority of parents in the US talk to their teenage children (13-17 years) about what is appropriate to share online (94%), and about online content that might be unsuitable (96%). Ofcom (2015) reports that in the UK 51% of parents of children 5-15 years talk to their children about sharing too much online, and 55% talk about content that might be unsuitable for their age. Overall, though, around 82% of UK parents of children 5-15 years state that they have talked to their children about managing online risks (Ofcom, 2015).

Parents can also restrict children’s ability to access specific online content or websites or set limits on the time they can spend online or using devices, and available data suggests this is more common in the US than in the UK. Just over half of parents in the US (55%) state that they have limited the amount of time, or time of the day, their teenage children (13-17 years) can go online (Anderson, 2016). In the UK, Ofcom (2015) reports that 32% of parents of children 8-11 years and 22% of parents of children 12-15 years have rules that determine ‘when and where’ children can go online. Although not referring to it directly, this rule is likely to affect the amount of time children spend online. With respect to the use of technical restrictions, Anderson (2016) finds that around 40% of parents of teenagers (13-17 years) in the US used parent controls to block or monitor teens’ online activities. Around one quarter (26%) of UK parents of children 5-15 years report using content filtering software or ISP network-level home filtering (Ofcom, 2015). Around one fifth of parents of children 5-15 years in the UK (18%) report using parent controls in device settings, but only 6% report using software that can limit the time children can spend on the internet (Ofcom, 2015). The specific indicators vary across both countries, as do the age groups covered, but broadly the available data suggest that the use of restrictions to mediate children’s internet use is lower in the UK than in the US.

Decisions parents make in relation to children’s screen time and use of technology reflect and intersect with national regulations and guidance, which may lead to cross-national differences (Hasebrink et al., 2009). There are no notable differences in the regulatory framework governing access to, and use of, the internet and technology in both countries. Public concern about the negative impact of children’s use of the internet and technology has been prominent in the UK and the US, but the UK, unlike the US, does not have official medical guidelines on the time children should spend in screen-based activities (Blum-Ross & Livingstone, 2016). This may account for the differences in parent reports of restricting children’s time using technology noted above. To the extent that such guidelines influence parents’ decisions about restricting screen time, and in turn children’s activities, we might expect US children to report less screen time and time using devices than their counterparts in the UK.

As with access to technology, socio-economic status is associated with parents mediating and restricting their children’s screen time; parents in higher socio-economic groups report that they exercise more control over children’s screen time (Livingstone & Helsper, 2008; Nathanson, 2001). Therefore, although children in more advantaged households might have more access to devices and the internet, parental mediation and restrictions in these families may counteract the potential for increased screen time. In contrast, although having less access to technology and the internet at home, low SES children may display more screen time because their parents place fewer restrictions on it. Educated parents may be more receptive to expert guidance and they have been shown to spend more time in active discussions with their children (Lareau, 2003). Time use research shows that children in higher socio-economic groups spend less time in screen-based activities such as watching TV and playing videogames (Hofferth, 2010; Mullan, 2018), suggesting that parental controls exert a stronger influence on children’s screen time than differential access. Educational levels are similar in both countries: 44% of US adults 25-64 have completed a tertiary degree, compared with 42% in the UK (Morshed, 2017); thus, although education is likely to influence children’s use of technology and screen time, we do not expect this to significantly moderate differences between the US and UK.

Parental ability to check and set limits on children’s online time will vary depending upon the time parents have at home with their children and data show cross-national differences in maternal employment patterns. In 2019, 72% of US mothers with children under 18 were in the work force, similar to 75% of UK mothers. However, 76% percent of those mothers were employed full time in the US, compared with only 50% in the UK (BLS, 2021; ONS, 2019). Research in the UK has shown that children with a full-time working mother spend more time watching TV and playing video games and less time using a computer (Mullan, 2018), but findings relating to mother’s employment are mixed across countries (Gracia et al., 2019). To the extent that parental monitoring leads to more restrictions on children’s screen time, we might expect UK children to report less time watching TV and playing video games than US children.

Further characteristics of the family that may influence children’s screen time and time using technology are the number of co-resident parents and the number of children in the family. Lone parents may find it more difficult to monitor children’s screen time compared with families with two co-resident parents. With respect to the influence of family structure on children’s daily time use, prior research suggests that the number of parents in the household has little impact on children’s screen time (Hofferth, 2010; Mullan, 2018, 2019), and there may be offsetting links between family structure, technology access, and parental mediation/restrictions in relation to screen time. The proportion of children in the US and the UK living in sole parent families is similar (23% and 21% respectively; Kramer, 2019). We have, therefore, no strong expectation about UK-US differences in the influence of lone-parenthood on children’s screen time and time using devices. With respect to number of children, US families are larger than in the UK with an average of 1.9 compared with 1.7 children (BLS, 2020; ONS, 2020). As with socio-economic status, the possible influence of family size on individual children’s screen time and time using devices is not straightforward. On the one hand, more children may limit access to devices and the internet for each individual child. On the other hand, it may be more difficult to monitor and restrict children’s overall screen time in larger than in smaller families. Despite this ambiguity, it is possible that family size differences will impact on cross-national patterns in children’s time in this domain in the US and the UK.

Although parents can, to an extent, control children’s time use, children and young people exercise agency in making decisions about their time use and can resist parental rules about screen time (Evans et al., 2011). No doubt reflecting this, younger children face more parental mediation and controls on their screen time than do adolescents (Nikken & Jansz, 2014). With growing independence and responsibility, older children are more likely to own smartphones (Mascheroni & Ólaffson, 2016), and children’s time using devices increases with age (Mullan, 2018; Mullan, 2020). Child gender is also significantly associated with different aspects of children’s screen time and time using devices. Most substantially, boys spend significantly more time playing video games than girls do (Hofferth & Moon, 2011; Mullan, 2018). Girls may not like playing videogames as much (see Hartmann & Klimmt, 2006), though it is also the case that parents mediate girls’ screen time more stringently than that of boys (Nikken & Jansz, 2014). Little is known about whether age and gender have similar influences on children’s screen-based activities and time using devices in the UK and the US. If parental control is higher in the US than the UK, it may be that cross-national differences are stronger among younger children and among girls.

Societal norms around what is appropriate concerning children’s time use can affect children’s capacity and freedom to make decisions about how they spend time. Prior research on cross-national differences in children’s time use has posited that children will spend more time in individually-oriented leisure activities and screen time in societies where individualism is highly valued (Gracia et al., 2019; Larson & Verma, 1999). The UK and the US are typically considered as exemplars of individualistic societies, and extant research shows that children in these countries do spend more time in screen-based activities than those in more collectivist societies (Gracia et al., 2019; Larson & Verma, 1999). To the extent, therefore, that individualism plays a decisive role in explaining cross-national patterns in children’s screen time and time using technology, we may expect similar patterns in the US and the UK. We have already noted, however, that family size is larger on average in the US, which may serve as a counterweight to individualism in the US in comparison with the UK.

Rapid technological change has altered the ways in which children use technology and engage with screens in their everyday lives. This has necessitated the collection of new kinds of data about the time children spend in these domains. Drawing on data from two innovative surveys that collect information about time in screen-based activities and time using devices in the UK and the US, this paper presents for the first time a comparative analysis of UK and US children’s daily time spent in these domains.

## Methods

### Data and Sample

Data on the time use of children 8-17 years in the UK are drawn from the latest UK Time Use Survey 2014-15 (hereafter UKTUS), which contains a representative sample of individuals aged 8+ years in UK households (Gershuny & Sullivan, 2017). Data on children’s time use in the US are from the Panel Study of Income Dynamics (PSID) Child Development Supplement 2014 (hereafter CDS). The CDS contains information on each child aged 0-17 years from families that completed a Core PSID interview in 2013, comprising a representative sample of children in the US (excluding those from families where both parents emigrated to the US after 1997) (Sastry & Fomby, 2017).

Both surveys collected activity data on a randomly sampled weekday and weekend day, using a 24-hour time diary instrument in which respondents provide information in their own words about their primary (or main) activities and any secondary activities. In describing their day children report their time in screen-based activities such as watching TV, playing videogames and using computers. A key innovation in the time diary instruments used in both surveys is that they include a field for children to report if they were using a device (such as a smartphone or tablet) at any point during the day alongside reports of their activities. This new field provides crucial extra information about the time children use devices which can overlap with time in other activities (both screen-based and non-screen activities) throughout the day. Although they are self-reported, previous research has demonstrated high reliability for diary-collected time use (Gershuny, 2000; Hofferth et al., 2008; Juster & Stafford, 1985; Robinson & Godbey, 1997).

In the UKTUS, children could report time using computers, smartphones or tablets, whereas in the CDS children could report using a wider array of devices (including TVs, video game consoles, and e-readers). To maximise the comparability of measures of children’s time using a device in both countries we ignore any information CDS children (in the US) provided about devices other than computers, smartphones and tablets. The wording used for the device field differed across surveys. UKTUS asked respondents about each 10-minute time period: *Did you use a smartphone, tablet, or computer?* The CDS instrument, which is activity focused, uses the following wording: *What did you use to watch or do the activity?* The latter explicitly links the use of a device to an activity (primary and/or secondary), whereas the former does not. The measure of activity time while using a device (smartphone, tablet, or computer) may therefore be less comprehensive in the US than in the UK. Our discussion of study limitations reflects on possible implications.

We conduct our analysis on a subsample of children 8-17 years available from each of these surveys. There are 3,852 diary days (UK: 2,186; US: 1,666) for children 8-17 years. We drop 107 diary days with more than four hours of missing time in the diary from our analysis (UK: 46; US: 61). We further omit 309 diary days due to missing data on the independent variables in the final analysis (UK: 94; US: 215). See Table 1 for a description of the characteristics of the final analysis sample.

**Table 1:** Sample characteristics, descriptive statistics: UK and US

	UK		US	
	*N*	*%*	*N*	*%*
Total (diary days)	2038	100.0	1390	100.0
Non-school day	1290	63.3	800	57.6
School day	748	36.7	590	42.4
8-10 years	627	30.8	457	32.9
11-13 years	586	28.7	409	29.4
14-17 years	825	40.5	524	37.7
Boy	1039	51.0	745	53.6
Girl	999	49.0	645	46.4
Mother not in paid work	633	31.0	389	28.0
Mother employed part-time	706	34.6	295	21.2
Mother employed full-time	699	34.3	706	50.8
Two-parent family	1636	80.3	1099	79.1
Lone-mother family	402	19.7	291	20.9
Mother no degree	1396	68.5	930	66.9
Mother degree	642	31.5	460	33.1
1st income quartile	505	24.8	342	24.6
2nd Income quartile	469	23.0	354	25.4
3rd income quartile	548	26.9	346	24.9
4th income quartile	516	25.3	349	25.1
One child 0-18 years	495	24.3	340	24.5
Two children 0-18 years	947	46.5	583	42.0
Three children 0-18 years	369	18.1	320	23.0
Four or more children 0-18 years	227	11.1	147	10.6
Internet at home	2006	98.4	1267	91.2
No internet at home	32	1.6	123	8.8

Weights applied

### Measures of Time Using Devices and Time in Screen-Based Activities

Our key outcomes of interest in this study relate to the time children spend using devices and time in screen-based activities throughout the day. With respect to the former, we construct a number of measures relating to children’s time using devices (smartphones, tablets, computers). First, we construct a measure of total time using these devices. This time may overlap with time in screen-based activities, or it may overlap with time in other activities. To capture this distinction, in addition to measuring total time using devices, we decompose this time as follows:

1. Time using device during a screen-based activity (as either a primary or a secondary activity): TV; videogames; computers
2. Time using device during non-screen leisure activities: socialising and games; hobbies and arts; and sport
3. Time using device during (non-screen) non-leisure activities: eating; study and reading; housework and shopping; civic and religious activities; paid work; and travel

A second set of measures captures total time in screen-based activities recorded as either primary or secondary activities. These comprise minutes watching TV, playing videogames, and using computers, and finally total time in screen-based activities. Note that children’s time using computers is the only activity that appears simultaneously in the activity field (and thus measured as a screen-based activity) and in the device use field (and thus included in measures of time using devices) in both surveys.

### Research Design

Our first research question addresses whether there are differences between children in the UK and the US in the measures outlined above. To examine this, we conduct a descriptive bivariate analysis of UK and US children’s total time using devices, decomposing this into time when children are using devices during screen-based activities, during non-screen leisure activities, and during non-leisure activities. We provide a further descriptive decomposition of this time looking at children’s time in specific activities within these three broad activity domains. To address our second research question, we estimate multivariate regressions to examine the extent to which factors known to influence children’s screen time explain cross-national patterns. We estimate multivariate regression models of measures of children’s total time using devices, and using devices during screen-based, non-screen leisure, and non-leisure activities. We then, in the last component of our analysis, conduct a multivariate analysis of children’s time in three specific screen-based activities (TV, video games, and using computers) when these are reported by the child as their primary or secondary activity. We estimate all multivariate regression models using ordinary least squares, which is appropriate for the analysis of time use data (Stewart, 2013). We apply sample weights throughout our analysis.

### Independent Variables

Our key independent variable indicates if the child is from the US, with the UK serving as the reference category. We add a control indicating if the diary day is a school day or not and include an interaction with this and the country indicator to account for differences in the effect of day type in both countries. Child age is entered in the model as a categorical variable with three values: 1) 8-10 years [reference]; 2) 11-13 years; and 3) 14-17 years. To capture child gender, a binary variable is entered in the model identifying if the child is a girl, with boy as the reference category. To examine potential UK-US differences in the influence of these child-level characteristics we include interactions between age and country, and gender and country.

In subsequent models we include a set of covariates to test whether key parent, family, and household characteristics moderate UK-US differences in children’s screen time and device use. Maternal employment status is entered in the model as a categorical variable with three values: 1) mother not in paid work [reference]; 2) mother works part time; and 3) mother works full time. To capture family structure, the model includes an indicator identifying children living in a lone mother family, with two-parent family as the reference category. The model also controls for number of children 0-17 years in the family: 1) one [reference]; 2) two; 3) three; 4) four or more.

To test the influence of socio-economic status on UK-US differences in children’s screen time and time using devices, the models include indicators for mother’s education and family income. Mother’s education is entered into the model as a binary variable indicating if the child’s mother has a post-high-school degree, with no degree as the reference category. Family income is a categorical variable: 1) 1^st^ income quartile [reference]; 2) 2^nd^ income quartile; 3) 3^rd^ income quartile; and 4) 4^th^ income quartile. Lastly, we include a variable indicating if the household has access to the internet at home, with households that do have access (the majority) as the reference category.

Table 1 provides a descriptive overview of the final analysis sample with respect to these characteristics. With a few exceptions, the samples are comparable. Consistent with expectations, UK mothers are more likely to work part-time and are less likely to have three or more children than US mothers. UK families are also more likely to have access to the internet.

## Results

### Bivariate Analysis of UK and US Children’s Time Using Devices

Table 2 shows estimates of the average time UK and US children aged 8-17 years spent using devices (computers/tablets/smartphones) on non-school and school days. On each day type, US children spent significantly less time using devices such as smartphones and tablets than UK children (*−*33.7 minutes and *−*19.8 minutes on non-school and school days respectively). This difference is most pronounced for time using devices alongside time in screen-based activities (*−*18.4 mins and *−*10.2 mins on non-school and school days respectively), though this is significant on non-school days only. Differences for time using devices during non-screen leisure and non-leisure activities are smaller and significant only on non-school days. Overall, UK-US differences in time using devices are most pronounced on non-school days when children’s time use is less constrained.

**Table 2:** Average minutes using devices (computers/tablets/smartphones) on school and non-school days: children 8-17 years in the UK and US

	UK	US	*diff*
Non-school day			
Total time using a device (computers/tablets/smartphones)	180.3	146.6	−33.7^***^
Screen time and using a device	116.0	97.6	−18.4^***^
Non-screen leisure and using a device	28.6	22.0	−6.5^*^
Non-leisure activities and using a device	35.7	26.9	−8.8^**^
School day			
Total time using a device (computers/tablets/smartphones)	108.7	88.9	−19.8^*^
Screen time and using a device	59.0	48.8	−10.2
Non-screen leisure and using a device	18.1	12.6	−5.5
Non-leisure activities and using a device	31.6	27.5	−4.1

Weights applied; n=3,737; ^*^ p < .05; ^**^ p < .01; ^***^ p < .001

**Fig. 1:**
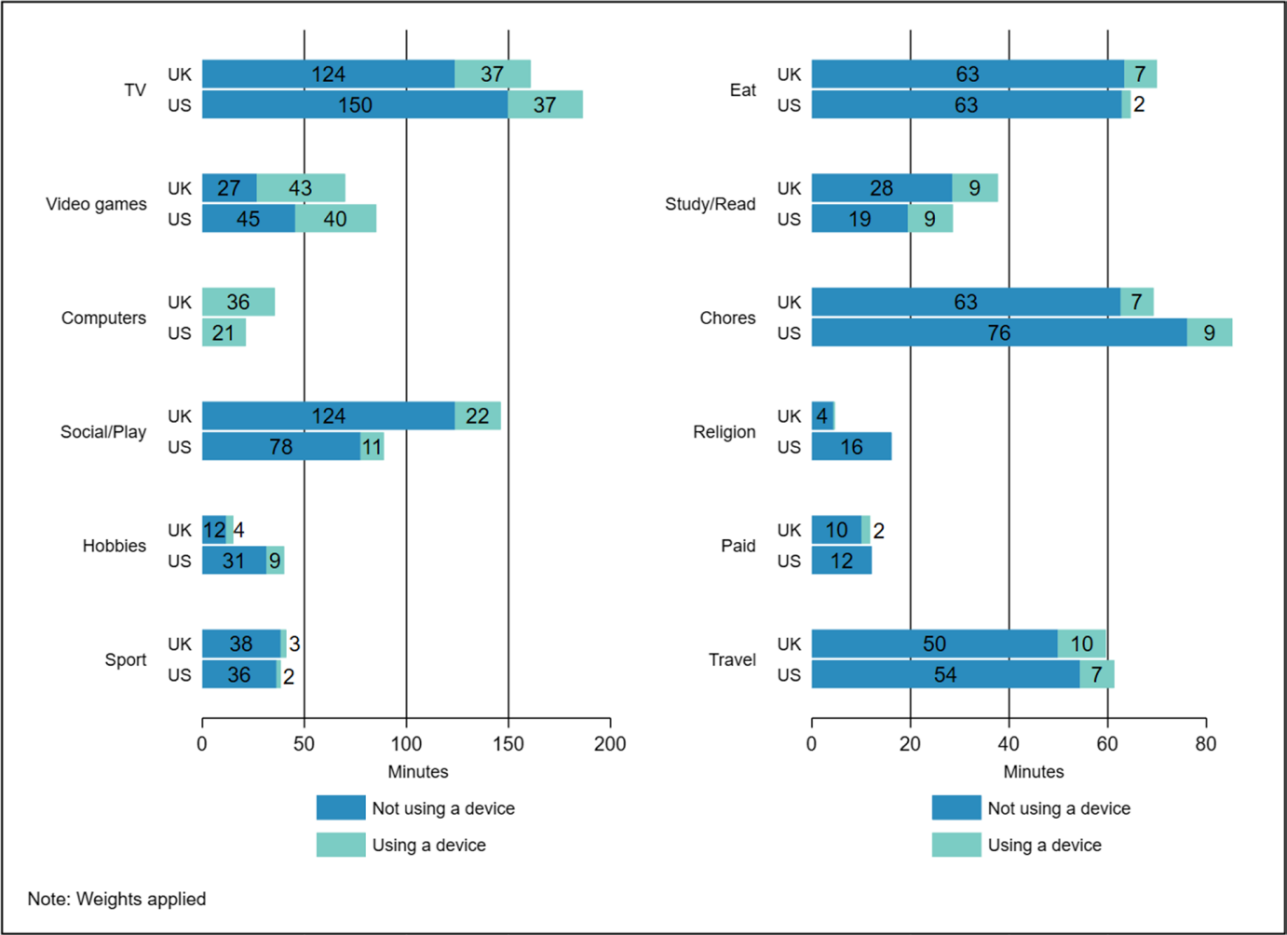
Average minutes using and not using devices during screen-based activities, non-screen leisure, and non-leisure activities: UK and US children 8-17 years, non-school days

**Fig. 2:**
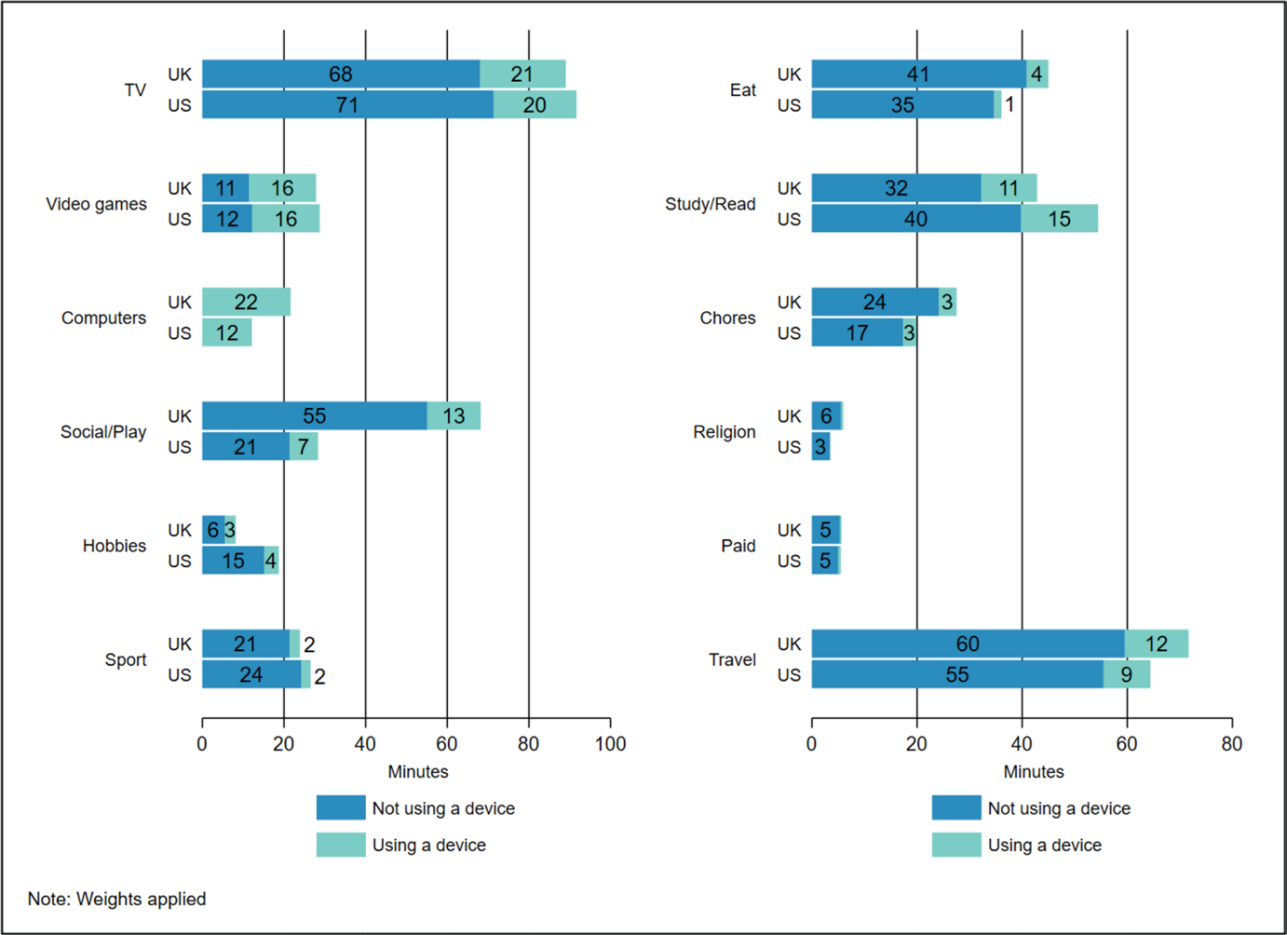
Average minutes using and not using devices during screen-based activities, non-screen leisure, and non-leisure activities: UK and US children 8-17 years, school days

Looking closer at these differences, Figures 1 and 2 show, for non-school and school days respectively, the average time UK and US children spent not using a device and using a device during screen-based activities (TV, videogames, and using computers), non-screen leisure activities (socialising/play, hobbies, and sport), and non-leisure activities (eating, studying/reading, chores, religious activities, paid work, and travel).

The UK-US difference in time using a device during screen-based activities (see Table 2) is concentrated in time when they report using computers as an activity. On both non-school and school days, UK children spent nearly twice as much time as US children using computers as an activity (see Figures 1 and 2 respectively). This makes up a large part of the overall difference in time using devices during screen-based activities, with minor difference in the time UK and US children spent using devices when also reporting watching TV or playing videogames. In total, though UK and US children spent a comparatively similar amount of time on average watching TV and playing videogames on school days (Figure 2), US children spent more time watching TV or playing videogames on non-school days (see Figure 1). Section 4.3 examines these differences more closely.

Looking at non-screen leisure activities, UK children spent more time using devices during social activities and play, and substantially more time in these activities in total, on both non-school and school days. On school days this difference is a result of US children spending significantly more time at school (school-related travel time is similar in both countries), with further analysis (results not reported) revealing that children in the US start school earlier than children in the UK. It is unlikely that this is a factor underpinning cross-national differences in time using devices, however, as cross-national patterns in time using devices are remarkably similar on non-school days. Regardless of whether the day is a school day or not, children in the US spent less time in social activities and play, and when they have more free time (as on non-school days) they spent this in other leisure activities. Notably, they spent more time in hobbies and more time using a device during this activity on non-school days.

The total difference in children’s time using devices during non-leisure activities (see Table 2) breaks down into comparatively minor differences across a wide range of activities including time eating, doing chores, and travelling (Figures 1 and 2). Broadly, these differences reflect cross-national differences in the total time children spent in these activities, with UK children spending more time eating and travelling. They also spent more time studying/reading, but this was the case on non-school days only. On school days, a reverse pattern was found with US children spending more time in this activity.

To summarise, cross-national differences in time using devices during screen-based activities are concentrated in time when children report using computers on both school and non-school days. Most time using devices during non-screen leisure is when children report social activities and play, and UK-US differences in device use are large during this time on both school and non-school days, offset partly on non-school days as US children spent more time using devices during hobbies. Differences in time using devices during non-leisure activities were comparatively small with no pronounced cross-national pattern across activities.

### Multivariate Analysis of Time Using Devices

Table 3 reports regression results for models of children’s time using devices controlling for country, school day, child age and gender, and interactions between country and the latter three variables. Results show that after controlling for child age and gender, there remained a significant country difference in total time using devices with US children spending 28.5 fewer minutes using devices in total. Echoing the bivariate analysis, this difference was concentrated in time during screen-based activities (−20.4 mins), though it was not statistically significant. There was also no significant UK-US difference found in children’s time using devices during non-screen leisure and non-leisure activities. Reflecting the bivariate analysis, the school day*country interactions are positive indicating that UK-US differences are lower on school days than on non-school days.

**Table 3:** OLS coefficients from models of minutes using devices: UK and US children 8-17 years

	Time using a device (smartphone, tablet, computer)			
	Total	During screen-based activity	During non-screen leisure	During non-leisure activity
US	−28.5^*^	−20.4	−6.3	−1.9
School day	−69.6^***^	−56.6^***^	−10.0^***^	−3.0
School day*US	13.8	8.7	0.9	4.1
11-13 years	60.6^***^	31.9^***^	8.9^**^	19.8^***^
14-17 years	128.9^***^	59.6^***^	21.7^***^	47.6^***^
11-13 years*US	−19.8	−11.3	−1.3	−7.3
14-17 years*US	−15.6	−15.3	6.1	−6.4
Girl	−26.6^***^	−45.1^***^	6.9^*^	11.6^***^
Girl*US	20.3	24.7^*^	−3.1	−1.3
Intercept	122.1^***^	104.3^***^	13.5^***^	4.3^*^
Adjusted R-square	0.15	0.10	0.05	0.10

Weights applied; n=3,737; ^*^ p < .05; ^**^ p < .01; ^***^ p < .001

The models show that total time using devices, and time using devices across all activity domains, increases substantially with age, with no significant difference in this association between the UK and the US (modest interaction effects are statistically insignificant). Girls spent significantly less time using devices during screen-based activities and spent significantly more time using devices during non-screen leisure and non-leisure activities. The interaction between sex and US was substantial and significant in the models for device use during screen-based activities (Table 3). In both countries, therefore, girls spent less time than boys using a device during screen-based activities, but this difference was significantly greater in the UK than in the US.

Table 4 shows the results from models with family level characteristics added. Subsequently, the UK-US difference in the total time children spent using devices was reduced by 28% and was no longer significant. There was a 38% reduction in the UK-US difference in time using devices during screen-based activities. Of all family-level factors added to the model, the number of children in the family and internet connection at home had the most significant impacts on children’s total time using devices and time using devices during screen-based activities. Having more siblings decreased the time children spent using devices during screen-based activities (and total time using devices). Not having a fixed internet connection had a similar negative association with this time; it limited the time children spent using a device like a smartphone, a tablet, or a computer in total, but especially during screen-based activity and even non-screen leisure.

**Table 4:** OLS coefficients from models of minutes using devices: UK and US children 8-17 years

	Time using a device (smartphone, tablet, computer)			
	Total	During screen-based activity	During non-screen leisure	During non-leisure activity
US	−20.6	−12.7	−4.4	−3.5
School day	−66.7^***^	−55.0^***^	−9.4^***^	−2.2
School day*US	8.0	4.8	−0.5	3.7
11-13 years	56.0^***^	29.5^***^	7.5^**^	19.0^***^
14-17 years	123.9^***^	53.7^***^	23.2^***^	47.0^***^
11-13 years*US	−18.0	−12.4	−0.7	−4.9
14-17 years*US	−12.7	−10.4	5.7	−8.0
Girl	−25.3^***^	−42.5^***^	6.0^*^	11.2^***^
Girl*US	26.4	25.4^*^	−0.1	1.2
Mother PT	10.1	5.1	1.2	3.8
Mother FT	−0.1	−3.6	−2.2	5.7
Lone mother	7.3	0.5	4.5	2.3
Mother has degree	2.9	−3.2	−3.8	10.0^**^
2nd Quartile	−8.5	−12.8	5.5	−1.3
3rd Quartile	−6.2	−6.0	2.5	−2.7
4th Quartile	−18.9	−15.1	−0.2	−3.6
2 children 0-17 years	−5.6	−9.2	8.1^*^	−4.5
3 children 0-17 years	−20.6^*^	−13.1	0.0	−7.5
4+ children 0-17 years	−28.8^*^	−19.3^*^	−1.9	−7.7
No internet at home	−56.3^***^	−43.3^***^	−11.1^**^	−2.0
Intercept	134.7^***^	121.5^***^	8.7	4.5
Adjusted R-square	0.16	0.10	0.06	0.11

Weights applied; n=3,428; * p < .05; ** p < .01; *** p < .001

Family structure (couple versus lone mother household), mother’s employment, education, and household income had no significant impacts on children’s time using devices, with one exception. In the model for time using devices during non-leisure activities, children whose mother had a degree spent more time using a device during non-leisure activities than children whose mother did not have a degree. There was no clear pattern of association between family income and children’s time using devices. Children in the top income quartile spent less time using a device (−18.9 mins), particularly during time in screen-based activities (−15.1 mins), but these coefficients were not statistically significant.

We estimated a fully interacted model to explore any UK-US divergence in the associations between family-level factors and children’s time using devices (results available on request). There were few significant interaction effects relating to family-level factors in these models. The results suggest a UK-US difference in the influence of living in a lone-mother household. US children in lone-mother families spent more time using a device than those in two-parent families, but there was no such device time difference among UK children. Lastly, middle-income children (2^nd^ and 3^rd^ income quartiles) spent significantly less time using devices in total than children in the lowest income quartile in the US, but not in the UK.

### Multivariate Analysis of Time in Screen-based Activities

The analysis turns now to children’s time in screen-based activities. Earlier we summarized UK-US differences in children’s time in specific screen-based activities (see Figures 1 and 2). Table 5 shows the results from multivariate regressions on children’s time in screen-based activities (TV, videogames, computers, and the total time in these activities) adjusting for child and family-level characteristics. The multivariate results largely confirm the descriptive bivariate analysis above, highlighting that the included covariates do not substantially moderate UK-US differences in children’s time in screen-based activities. With the specification including an interaction between country and school day in the model, the main effect for country corresponds to non-school days and confirms that US children spent more time watching TV, but less time using computers than UK children. Children in both countries spent less time in total screen-based activities on school days compared with non-school days, but the interaction effect shows that the country difference reversed completely on school days, confirming the bivariate analysis above (Figure 2) showing no difference in time in screen-based activities on school days. The sole exception here relates to the model for time using computers, which confirms that US children spent significantly less time using computers on both school and non-school days.

**Table 5:** OLS coefficients from models of minutes in screen-based activities: UK and US children 8-17 years

	TV	Video games	Computers	Total screen-based activities
US	34.8^**^	5.3	−18.2^***^	21.9
School day	−71.7^***^	−42.1^***^	−13.9^***^	−127.6^***^
School day*US	−21.8^*^	−10.7	4.0	−28.6^*^
11-13 years	9.1	13.0^**^	7.9^*^	30.0^***^
14-17 years	17.4^**^	1.1	18.5^***^	37.0^***^
11-13 years*US	−1.3	0.3	−2.1	−3.1
14-17 years*US	−9.1	19.4	−5.5	4.8
Girl	4.8	−71.8^***^	−5.6	−72.6^***^
Girl*US	−7.6	−1.8	18.6^***^	9.2
Mother PT	6.5	1.7	−1.9	6.2
Mother FT	0.9	8.3	−7.7^*^	1.5
Lone mother	15.9^*^	−7.9	−2.1	5.9
Mother has degree	−24.8^***^	5.6	−2.4	−21.6^**^
2nd Quartile	1.9	0.3	−8.0	−5.8
3rd Quartile	8.8	−2.0	−2.1	4.7
4th Quartile	−7.9	−17.1^*^	−6.4	−31.4^**^
2 children 0-17 years	0.8	−3.3	−8.6^*^	−11.1
3 children 0-17 years	13.7	−0.7	−9.1^*^	3.9
4+ children 0-17 years	3.0	−11.6	−12.2^*^	−20.9
No internet at home	−0.5	−11.1	−8.2	−19.8
Intercept	145.7^***^	103.5^***^	44.0^***^	293.2^***^
Adjusted R-square	0.12	0.17	0.04	0.23

Weights applied; n=3,428; ^*^ p < .05; ^**^ p < .01; ^***^ p < .001

Children 11-13 years and adolescents 14-17 years spent significantly more time in screen-based activities than did children 8-11 years. Differences in screen-based activities between children 8-10 years and 11-13 years were concentrated in time playing videogames, whereas adolescents spent significantly more time watching TV than children 8-10 years. Children in both older age groups spent more time using computers than children in the youngest age group.

Girls spent significantly less time playing videogames than boys, which accounts for the gender difference in total time in screen-based activities, and this applied equally in the UK and the US. There were noteworthy cross-country differences in the time boys and girls spent using computers as a primary or secondary activity. Overall, there was no gender difference in time using computers (see Table 5), but the interaction with country and gender was significant indicating diverging results in each country in relation to gender. Predicted minutes estimated from the model show that in the UK, girls averaged slightly less time using computers than boys (26 mins vs. 33 mins) whereas in the US girls averaged more time using computers than boys (24 mins vs 13 mins). Consequently, cross-national differences in time using computers (greater in the UK) were concentrated among boys, with no significant difference found between girls in the UK and the US.

Children whose mother worked full-time spent significantly less time using computers than children in couple male-breadwinner households. This was an unexpected and puzzling result with no clear explanation (which we will return to when discussing results from fully interacted models below). With respect to factors linked to socio-economic status, children whose mother has a degree spent significantly less time in screen-based activities, which was concentrated in time watching TV, with no significant differences in time playing video games or using computers. With respect to income, children in top income quartile families spent significantly less time in total screen-based activities, particularly in time playing videogames, than children in the lowest income quartile families.

Children in larger families spent less time using computers (see Table 5), which may reflect limitations in accessing computers in larger families as it is unlikely that each child would have exclusive use of a computer. There was a comparable negative coefficient for time playing videogames though this was insignificant. Lastly, total time in screen-based activities was not significantly associated with access to internet connection at home.

As above, we estimated a fully interacted model to explore any UK-US divergence in the associations between family-level factors and children’s time in screen-based activities (results available on request). The results indicate that the negative association between full time maternal employment and children’s time using computers (see Table 5) was found only in the UK. In addition, UK children with mothers in full-time employment spent more time watching TV (though this was not statistically significant) and significantly more time playing videogames than those with mothers not in paid work. In contrast, maternal employment in the US had no significant influence on any aspect of children’s screen-based activities. Results for lone-motherhood also varied across countries. In the US, children in lone mother families spent significantly more time in screen-based activities (concentrated in time watching TV). In contrast, UK children in lone mother families spent significantly less time playing videogames, and less time in total screen-based activities though this latter result was not statistically significant.

Turning to factors capturing children’s socio-economic status, there were a few significant interactions between country and maternal education. The significant negative association between mother’s education and children’s total time in screen-based activities (see Table 5) was found in the UK only. Although mother’s degree status had a negative association with children’s time watching TV in both countries, US children with a mother who has a degree unexpectedly spent significantly more time playing videogames. There were no significant country interactions linked to income, number of children, or internet connection.

### Study Limitations

The analysis in this paper was possible due to the existence of two unique surveys that collected rich data on children’s time using devices and in screen-based activities, recognising that time in this area is now integrated into many different aspects of daily life. Although similar, the time diary instruments used by these surveys to collect time use data are not identical. As noted above, the US time diary prompted children to think directly about their primary and secondary activities when reporting device use, while UK children reported any device use per time point. The fact that US children report spending less time using devices overall may be an effect of the difference in wording used in the time diaries. This is less likely to influence screen time spent using a device, because in both countries a device is highly likely to be used for the activity. Significantly, it is in screen time that the difference is largest. Bear in mind also, that children in the US report less time using computers as a main or secondary activity (and this includes time using tablets or smartphones), and there is no difference in the wording used in the primary/secondary activity fields in the diary instruments in both countries. Therefore, the difference we find in time using devices such as computers is consistent across different components of the diary instruments. A limitation of both instruments is that neither collects detailed information about what children are doing with the devices. This could provide further insights into cross-national differences in children’s time in this domain. As should be now evident, time diary instruments can be adapted to collect data about children’s time using devices, thereby enhancing our understanding of children’s time in this critical area, and efforts should be taken to maximise the reliability and comparability of instruments used in different countries.

## Discussion and Conclusion

The time children and young people spend in activities linked to screens has changed irrevocably. Once the sole screen in children’s daily lives, the TV now competes with an array of other screen-based devices such as computers, smartphones, tablets, and videogame consoles. Enabled by increasingly fast and reliable internet connections, children are now incorporating screen-based devices into a wide range of daily activities. This has led to concern that children are spending an excessive amount of time in screen-based activities and using devices such as smartphones and tablets (Auxier et al., 2020; Kardefelt-Winther, 2017; Smahel et al., 2012). Running through these debates and concerns are questions about the actual amount of time children spend using screens. The pandemic pushed these debates to one side as children were forced to spend vastly increased amount of time online for education and other purposes. Nevertheless, concerns about children’s screen time persist and questions about the long-term consequences of the pandemic for children’s screen time will increase. Without reliable estimates of this time, in total and linked to or alongside time in other daily activities, ongoing debates are at risk of being untethered from the daily realities of children’s screen and device time use.

Time use surveys provide reliable estimates of time spent in various daily activities and are responding to the challenges of measuring screen time stemming from technological change. This paper analysed data from two surveys (from the UK and the US) where the time-diary instrument has been adapted to collect data on time using devices (smartphones, tablets, computers) alongside reports of time in daily activities. These data allow us to conduct, for the first time, a thorough comparative analysis of children’s time using devices throughout the day. We show that children in both countries are using devices such as smartphones, tablets, and computers alongside the time they spend in other activities including screen-based activities, non-screen leisure, and non-leisure activities. Overall, the time children spent using devices was higher in the UK than in the US. This UK-US difference was concentrated in time using a device during screen-based activities, whereas UK-US differences in children’s time using devices during non-screen leisure and non-leisure activities were comparatively modest and occurred only on non-school days.

The large cross-national difference in time using devices during screen-based activities was explained by two factors: differences in family size and differences in access to the internet across the two countries. Families in the US are, on average, larger and, consequently, may have to share devices across siblings, reducing ‘media multi-tasking’ (Rideout, 2016). Though most families with children have a home internet connection, the proportion is lower in the US than in the UK. Access to the internet is lower in the US for a number of important reasons, including its large geographic area and isolation of some communities, though the US has invested heavily to expand broadband access in recent years (Romm & Zakrzewski, 2021). It may be the case that these differences have diminished over time, a question for future research. Looking closer at the UK-US difference in time in screen-based activities using devices, however, we found that UK children spent more time using computers. Consistent with their lower use of devices, children in larger families spent less time using computers, which likely resulted from limits on access for any individual child to a personal computer or laptop in larger families. This suggests a heightened risk faced by children in larger families of limited access to computers at home, and this is more pronounced in the US that has larger families and higher poverty exposure than in the UK.

The negative gap in screen-based activities due to lower time using computers of children and young people in the US compared to the UK was more than offset by differences in time watching TV and playing videogames, particularly on non-school days. While US children are spending less time using devices, they are spending more time in established screen-based activities. Cross-national patterns in time in established screen-based activities differ markedly in two important respects from patterns observed in time using newer mobile devices. First, cross-national differences in total time using devices occur on non-school and, to a lesser extent, school days; differences in screen-based activities arise on non-school days only. Second, the UK-US difference in time watching TV remains significant after controlling for child and family characteristics. This suggests a comparatively stable preference for, and acceptance of, US children spending time watching TV, particularly on days when they have most free time. In the US, long-established screen-based activities exert a stronger pull on children’s attention in comparison with the UK, where children’s time is relatively more concentrated on newer devices.

Differences in access to devices and the internet help explain part of the cross-national difference in time using newer devices, but it cannot explain why US children spend more time watching TV, as opposed to using computers, as a main or secondary activity. This is despite the fact that the US, and not the UK, has professionally recommended guidelines for children’s screen time (though these directly apply to children younger than in this study). Our results show that UK children spend more time in social activities and games (on both school and non-school days) whereas US children spend more time in hobbies and arts activities (again on both school and non-school days). This may reflect differences in preferences for distinct kinds of leisure, or differences in opportunities for non-screen leisure activities, including outside the home. The results shown here point to the need to place children’s screen time in the wider context of their daily lives both in terms of other activities in which they engage and the social context of their daily activities.

Social norms and values may help explain cross-national differences in children’s time use (Gracia et al., 2019; Larson & Verma, 1999). The importance of family to US children is clear in the significant influence of family size on US children’s device use and computer use, even controlling for internet access, family income, and maternal education. It is unlikely, however, that this provides a complete explanation for the differences we find in children’s time in screen-based activities. Both countries are also notable for placing high social value on individualism. It is unlikely that differences in parental values and attitudes around children’s use of technology are a compelling factor here either (Hasebrink et al., 2009). Research suggests that parents in the US are more likely than in the UK to monitor young children’s screen use, but the documented differences occur primarily among older children. As Larson and Verma (1999) highlight, material factors such as access to transport, and leisure activities outside the home influence children’s daily time use. Concerns for their safety outside the home, moreover, may influence their time use. Whether there are salient cross-national differences in these respects is an open question. Gender remains an important distinguisher of children’s use of devices, with girls less likely to use devices such as videogame consoles, overall, but older boys appear to be the least likely to use computers, especially in the US. Thus, we cannot also ignore the preferences and decisions of children and young people themselves and there could be important cross-national differences in gender values that may account for the differences we observe in their daily time use patterns.

Our analysis also highlighted that mother’s employment status and family structure (lone-mother/two-parent family) had significant but contrasting influences in the UK and the US. In the UK, children with mothers employed full time spent more time in screen-based activities whereas the opposite was the case in the US. This aligns with prior research showing that associations between mother’s employment and children’s time use vary cross-nationally (Gracia et al., 2019) as does the fraction employed full-time. US children in lone-mother families spent more time watching TV than their counterparts in two-parent families, which was not the case in the UK. Cross-national differences in the social and economic context that provides support for working mothers and lone parents, including access to after-school childcare settings, may underpin the differences we observe in the association between these factors and children’s time use. We do not consider race and ethnicity in our analysis due to limitations in the UK data. Here we highlight the need to exercise caution in drawing conclusions about associations linked to these characteristics across diverse social and policy contexts.

The influence of socio-economic status is relatively consistent across countries, with the strongest and most consistent association linked to mother’s education. Net of educational level, income had a limited influence on children’s device and screen time in both countries. The recent pandemic and subsequent lockdown and closure of schools highlighted, in both the UK and the US, a stark socio-economic divide in access to computers and powerful home internet connection needed to engage fully with online learning. Our results show, prior to the COVID-19 pandemic, no significant association between children’s time using computers and indicators of socio-economic status (mother’s education and family income) in either the UK or the US. Before the pandemic, children were receiving education at school as normal, and the lack of access to technology and the internet at home was linked to less device use during screen-based activity, but socio-economic differences in time using computers outside school were not apparent. The COVID-19 pandemic has brought into sharp relief latent socio-economic differences in children and adolescent’s access to, and time using, computers and other technology essential to their education, particularly in the US (Horrigan, 2020).

The COVID-19 pandemic made navigation of daily life without screens and internet connectivity impossible, and there can be little doubt that children in both countries analysed in this paper spent substantially more time online and in screen-based activities during the pandemic period. Whether this has a long-lasting effect on children’s time in screen-based activities and using devices in the UK, the US, and elsewhere will be a topic for future research and debate. This paper provides a critical baseline from which to compare future trends in both countries and serve as a useful comparison for other countries. It adds to an emerging literature exploring cross-national differences in children’s time use. The focus here was on the time children spend using devices and in screen-based activities, revealing the extent to which children’s daily use of technology overlaps with many of their daily activities in the UK and the US. Although single catch-all measures of children’s time engaging with screens may be useful, it is vital not to lose sight of the fact that time in this domain has diversified, and, as we have seen, varies substantially across countries similar in many key respects. As more countries adapt time-diary instruments to collect reliable data on time spent using devices we can look forward to extending our understanding of the influence of technological change and potential long-term impacts from the pandemic on children’s daily time use patterns.

## Footnotes

1 https://www.oecd.org/social/income-distribution-database.htm
